# Case Report: Successful hematopoietic stem cell transplantation in pediatric pyruvate kinase deficiency: a single-center Asian case series demonstrating favorable outcomes

**DOI:** 10.3389/fimmu.2026.1831203

**Published:** 2026-08-24

**Authors:** Haomin Yan, Dongjun Li, Xiaoxi Lu, Xue Yang, Yiping Zhu, Shuwen Sun

**Affiliations:** 1 Department of Pediatrics, West China Second University Hospital, Sichuan University, Chengdu, China; 2 Key Laboratory of Obstetric and Gynecologic and Pediatric Diseases and Birth Defects, Sichuan University, Ministry of Education, Chengdu, China

**Keywords:** allogeneic hematopoietic stem cell transplantation, anti-thymocyte globulin (ATG), graft-versus-host disease, myeloablative conditioning, pyruvate kinase deficiency, thiotepa

## Abstract

Pyruvate kinase deficiency (PKD) is a rare autosomal recessive inherited hemolytic anemia caused by pathogenic variants in the PKLR gene. The disease exhibits marked clinical heterogeneity, ranging from compensated anemia to severe transfusion-dependent phenotypes presenting early in childhood. Although supportive care and emerging pharmacologic agents such as pyruvate kinase activators have improved disease management, treatment options remain limited for patients with severe, transfusion-dependent disease. Allogeneic hematopoietic stem cell transplantation (allo-HSCT) represents a potential curative approach. However, clinical experience in pediatric PKD remains limited, and reported outcomes are heterogeneous. In particular, data regarding standardized conditioning strategies, donor variability, and early post-transplant outcomes are still insufficient. Here, we report a single-center case series of six pediatric patients with genetically confirmed PKD who underwent allo-HSCT between 2019 and 2026. The cohort included both matched sibling and unrelated donors with varying HLA compatibility. All patients achieved successful hematopoietic engraftment, with neutrophil and platelet recovery occurring within 12–16 days post-transplantation. Complete donor chimerism (>98%) was achieved in all evaluable patients by day +21. No primary graft failure was observed, and no severe acute or chronic graft-versus-host disease occurred during early follow-up. Importantly, patients demonstrated rapid correction of transfusion dependence following transplantation. However, follow-up duration was heterogeneous, ranging from several months to over six years, and long-term outcomes remain under continued observation. This study provides a homogeneous real-world experience suggesting that allo-HSCT using a thiotepa- and ATG-containing myeloablative regimen is feasible in selected pediatric PKD patients, including those receiving unrelated donor grafts. These findings support further investigation of standardized transplant approaches and early intervention strategies in severe pediatric PKD. Larger multicenter studies with long-term follow-up are required to validate safety, durability, and late effects of transplantation in this rare disease.

## Introduction

Pyruvate kinase deficiency (PKD) is a rare autosome recessive congenital disorder caused by mutations in the *PKLR* gene. Pyruvate kinase acts as a key glycolytic enzyme in the conversion of phosphoenolpyruvate to pyruvate with concomitant production of ATP, on which mature red blood cells rely on for energy to maintain normal structure and function (1, 2). Defects in *PKLR* function affect the normal metabolism of erythrocytes and ultimately lead to a wide spectrum of hemolysis, from chronic mild compensated anemia, jaundice and asymptomatic gallstones to acute severe hemolysis that requires regular transfusions (3).

Treatments for pyruvate kinase deficiency have historically been limited to supportive care, periodic blood transfusion, iron chelation and splenectomy. Splenectomy partially ameliorates the hemolytic anemia and decreases the transfusion burden in most patients and increases the hemoglobin by a median of 1.6 g/dL (4). The genotype of *PKLR* mutation correlates with clinical manifestation, as patients with two non-missense mutations have lower hemoglobin levels and higher complication rates (5). Most patients have at least one missense *PKLR* mutation (6), and the common missense variants include the R479H mutation in Amish communities (7). Mitapivat, an oral allosteric pyruvate kinase activator, has introduced disease-modifying options, which is already approved in the USA for the treatment of PKD in adults (8, 9), and the pediatric clinical trials are underway.

Allogeneic hematopoietic stem cell transplantation (allo-HSCT) is currently the only potentially curative therapeutic option for PKD, as it can restore normal erythropoiesis through complete donor hematopoietic reconstitution (10, 11). Nevertheless, due to the rarity of the disease, published pediatric experience remains limited, and existing evidence is mainly derived from isolated case reports or small heterogeneous series. Available literature suggests that both peripheral blood stem cell and bone marrow transplantation have been used in PKD (12), most commonly with myeloablative conditioning regimens (13). Reported complications include graft-versus-host disease (GVHD), infections, and graft failure. However, outcomes remain inconsistent, and comparative data across donor types and transplant strategies are still lacking.

Here, we report a single-center case series of six pediatric patients with genetically confirmed PKD who underwent early allo-HSCT using a uniform and intensified myeloablative conditioning regimen consisting of cyclophosphamide, busulfan, fludarabine, thiotepa, and anti-thymocyte globulin. Notably, transplantation was performed at an early stage of disease course before the development of advanced transfusion-related complications in most patients, highlighting the potential benefit of timely intervention. A distinctive feature of this cohort is the application of a standardized conditioning and transplant protocol across all patients, which incorporates our optimized preconditioning strategy and allows for a more consistent evaluation of transplant outcomes across heterogeneous donor sources, including both matched sibling and unrelated donors, as well as different GVHD prophylaxis approaches. This study aims to provide a homogeneous real-world dataset to complement existing literature and to contribute to a more standardized understanding of transplant feasibility, engraftment kinetics, and early post-transplant outcomes in pediatric PKD.

## Case description

### Patient 1

A 2-year and 11-month-old male patient was admitted to our hospital on July 3, 2019. Three hours after birth, he developed generalized jaundice, pallor, tachycardia, and reduced responsiveness. He later developed recurrent anemia and jaundice and was diagnosed with congenital pyruvate kinase deficiency by genetic testing, carrying compound heterozygous PKLR mutations (maternal c.1343G>A, paternal c.1436G>A) (**Table 1**). From 2 months of age, he required regular red blood cell transfusions approximately every 30 days to maintain stable hemoglobin levels (7-8g/dL). Long-term transfusion resulted in iron overload, with a pre-transplant serum ferritin level of 2243.6ng/ml. He received iron chelation therapy with deferasirox (250 mg twice daily). No splenomegaly or other PKD-related complications, such as gallstones or severe growth retardation, were observed before transplantation.

**Table 1:** Sequencing results of the four patients and their parents.

Patient	*PKLR* gene mutation	Origin	Nucleotide substitution	Amino acid substitution	Exon	Mutation type
Patient 1	heterogenous	Paternal	c.G1529A	p.R510Q	exon10	missense mutation
		Maternal	c.G1436A	p.R479H	exon 9	missense mutation
Patient 2	homozygous	Paternal	c.941T>C	p.I314T	exon 6	missense mutation
		Maternal	c.941T>C	p.I314T	exon 6	missense mutation
Patient 3	heterogenous	Paternal	c.1468C>T	p.R490W	exon 10	missense mutation
		Maternal	c.1178A>G	p.N393S	exon 8	missense mutation
Patient 4	heterogenous	Paternal	c.1675C>T	p.Arg559Ter	exon 11	nonsense mutation
		Maternal	c.353A>G	p.Asn118Ser	exon 3	missense mutation
Patient 5	heterogenous	Paternal	c.1607G>A	p.G536D	exon 10	missense mutation
		Maternal	c.1126A>C	p.S376R	exon 8	missense mutation
Patient 6	heterogenous	Paternal	c.730G>A	p.G244R	exon 6	nonsense mutation
		Maternal	c.1054C>T	p.P352S	exon 8	missense mutation

Prior to HSCT, the patient received a myeloablative conditioning regimen consisting of cyclophosphamide, busulfan, fludarabine, thiotepa, and anti-thymocyte globulin. He subsequently received G-CSF mobilized peripheral blood stem cells from an unrelated 10/10 HLA-matched donor, with CD34+cells at 4.55×10^6/kg, total nucleated cells at 9.97×10^8/kg, and CD3+ cells at 39×10^7/kg. GVHD prophylaxis included mycophenolate mofetil, methotrexate, and cyclosporine. Neutrophil and platelet engraftment occurred on day +16 and day +13, respectively. Donor chimerism on day +21 was 99.56%. The patient was discharged on day +31 (August 20, 2019) and has been followed regularly for more than 83 months, with good clinical status (**Tables 2**, **3**).

**Table 2:** Laboratory characteristics of the patients before and after HSCT.

Time	Characteristics	Patient 1	Patient 2	Patient 3	Patient 4	Patient 5	Patient 6
Before HSCT							
	Whole blood count						
	RBC (×10^12^)	2.88	2.77	3.16	3.00	2.34	2.41
	Hemoglubin (g/dL)	8.5	8.0	9.3	8.6	7.7	7.5
	Biochemistry						
	ALT (U/L)	18	33	19	34	67	80
	AST (U/L)	48	49	29	80	70	91
	TBIL (umol/L)	51.7	88.0	64.1	104.9	159.4	60.3
	IDBL (umol/L)	41.1	81.1	51.6	86.1	144.6	50.8
	Ferritin(ng/ml)	2243.60	1460.60	1368.80	1086.00	582.70	1191.50
1 month after HSCT							
	Whole blood count						
	RBC (×10^12^)	4.14	2.98	3.60	2.74	3.45	3.39
	Hemoglubin (g/dL)	12.5	9.5	11.2	8.5	10.5	10.8
	Biochemistry						
	ALT (U/L)	23	60	22	118	10	42
	AST (U/L)	44	109	29	88	24	68
	TBIL (umol/L)	7.1	10.7	12.1	13.3	15.8	13.0
	IDBL (umol/L)	4.8	7.4	9.4	10.1	7.0	8.2
	Ferritin(ng/ml)	3699.20	7349.40	2612.70	5177.00	3587.40	>1650
3 months after HSCT							
	Whole blood count						
	RBC (×10^12^)	3.22	3.35	3.32	3.48	3.68	3.32
	Hemoglubin (g/dL)	10.7	11.2	10.9	12.1	12.4	99
	Biochemistry						
	ALT (U/L)	23	29	19	39	45	27
	AST (U/L)	45	53	48	83	29	49
	TBIL (umol/L)	5.1	7.9	8.6	10.8	8.8	5.5
	IDBL (umol/L)	3.0	4.8	6.0	8.2	5.7	3.6
	Ferritin(ng/ml)	1993.70	2246.50	1427.70	10493.30	2334.50	4695.70
1 year after HSCT							
	Whole blood count						
	RBC (×10^12^)	3.71	3.95	4.15	4.53	NA^*^	NA
	Hemoglubin (g/dL)	11.9	11.9	13.3	13.2	NA	NA
	Biochemistry						
	ALT (U/L)	27	35	23	36	NA	NA
	AST (U/L)	46	48	31	43	NA	NA
	TBIL (umol/L)	7.1	9.3	13.8	11.3	NA	NA
	IDBL (umol/L)	4.6	6.0	9.8	9.6	NA	NA
	Ferritin(ng/ml)	784.00	1032.20	625.20	1480.10	NA	NA

*The HSCT of patient 5, 6 was conducted on 8 January 2026 and 11 March 2026.

**Table 3:** Details of transplantation.

Characteristics	Patient 1	Patient 2	Patient 3	Patient 4	Patient 5	Patient 6
Sex	Male	Male	Female	Female	Male	Male
Age at HSCT	2.9 years	2.3 years	4.3 years	3.3 years	9.9 years	3.3 years
Stem cell source	PBSC	PBSC	PBSC	PBSC	PBSC	PBSC
Donor	unrelated	sibling	unrelated	unrelated	unrelated	unrelated
HLA match	identical	identical	identical	9/10	identical	9/10
Follow-up time(month)	83	27	22	13	6	3
Outcome	alive	alive	alive	alive	alive	alive

### Patient 2

A 2-year and 3-month-old male patient was admitted on February 26, 2024. He developed jaundice at 2 months of age, and hemoglobin decreased to a nadir of 5g/dL. Genetic testing confirmed congenital pyruvate kinase deficiency with a homozygous PKLR mutation c.941T>C (p.I314T) (**Table 1**). From 2 months of age, he required regular red blood cell transfusions every 28–30 days. Pre-transplant serum ferritin was 1460.60ng/ml, and no iron chelation therapy was administered. Physical examination and CT imaging revealed no splenomegaly. No other PKD-related complications, such as gallstones or significant growth retardation, were observed. Prior to HSCT, the patient received a myeloablative conditioning regimen consisting of cyclophosphamide, busulfan, fludarabine, thiotepa, and anti-thymocyte globulin. He subsequently received G-CSF mobilized peripheral blood stem cells from his biological brother (12/12 HLA-matched sibling donor), with CD34+ cells at 6.76×10^6/kg, total nucleated cells at 6.79×10^8/kg, and CD3+ cells at 2.36×10^8/kg. GVHD prophylaxis consisted of cyclosporine, mycophenolate mofetil, and methotrexate. Neutrophil and platelet engraftment occurred on day +14 and day +13, respectively. Donor chimerism on day +21 was 99.58%. The patient was discharged on day +35 and has been followed for more than 27 months (**Tables 2**, **3**).

### Patient 3

A 4-year and 3-month-old female patient was admitted on August 24, 2024. She developed recurrent jaundice and anemia shortly after birth and was diagnosed at 4 months of age with congenital pyruvate kinase deficiency carrying compound heterozygous PKLR mutations (maternal c.1178A>G, paternal c.1648C>T) (**Table 1**). From 6 months of age, she required regular red blood cell transfusions approximately once per month. Pre-transplant serum ferritin was 1368.8ng/ml, and iron chelation therapy with deferasirox was initiated 6 months before HSCT. Physical examination revealed splenomegaly: Kaplan line I 8 cm, line II 9 cm, and line III -5 cm below the costal margin. The spleen was of moderate consistency with a rounded edge, and no prior splenectomy had been performed. No other PKD-related complications, such as gallstones, were observed. She received a myeloablative conditioning regimen consisting of cyclophosphamide, busulfan, fludarabine, thiotepa, and anti-thymocyte globulin. She subsequently underwent transplantation from a 10/10 HLA-matched unrelated donor peripheral blood stem cell graft, with CD34+ cells at 6.30×10^6/kg, total nucleated cells at 8.49×10^8/kg, and CD3+ cells at 6.23×10^7/kg. GVHD prophylaxis included methotrexate, cyclosporine A, and mycophenolate mofetil. Neutrophil and platelet engraftment occurred on day +12 and day +16, respectively. Donor chimerism on day +18 was 99.82%. The patient was discharged on day +29 and has remained well during 21 months of follow-up (**Tables 2**, **3**).

### Patient 4

A 3-year and 3-month-old female patient was admitted on May 23, 2025. She presented with persistent pallor and jaundice after birth and was diagnosed with congenital pyruvate kinase deficiency carrying compound heterozygous PKLR mutations (maternal c.353A>G, paternal c.1675C>T) (**Table 1**). She had severe anemia with hemoglobin levels of 6–7 g/dL and required regular red blood cell transfusions approximately every 6 weeks to maintain stable hemoglobin levels. Pre-transplant serum ferritin was 1086.0ng/ml, and no iron chelation therapy was administered. Physical examination and CT imaging revealed no splenomegaly. No other PKD-related complications, such as gallstones or growth retardation, were observed. She received a myeloablative conditioning regimen consisting of cyclophosphamide, busulfan, fludarabine, thiotepa, and anti-thymocyte globulin. She subsequently underwent transplantation from a 9/10 partially matched unrelated donor peripheral blood stem cell graft, with CD34+ cells at 5.78×10^6/kg, total nucleated cells at 10.77×10^8/kg, and CD3+ cells at 19.25×10^7/kg. GVHD prophylaxis included methotrexate, cyclosporine A, and mycophenolate mofetil. Neutrophil and platelet engraftment occurred on day +12 and day +11, respectively. Donor chimerism on day +21 was 98.78%. The patient was discharged on day +34 (**Tables 2**, **3**).

### Patient 5

A 9-year and 10-month-old male patient was admitted on December 20, 2025. He presented at birth with fatigue, pallor, jaundice, and dark urine with hemoglobin <3 g/dL. He initially required blood transfusion about every 3 months, but transfusion was discontinued after 1 year due to family decision, with hemoglobin levels at 4.9-6.0 g/dL. Genetic testing confirmed congenital pyruvate kinase deficiency with compound heterozygous PKLR mutations (paternal c.1607G>A p.G536D, maternal c.1126A>C p.S376R) (**Table 1**). Pre-transplant serum ferritin was 582.7ng/ml, with no iron chelation therapy administered. Physical examination revealed splenomegaly: Kaplan line I 5.5 cm, line II 4.5 cm, and line III-1.0 cm below the costal margin. The spleen was soft, and no prior splenectomy had been performed. No other PKD-related complications were observed. He received a myeloablative conditioning regimen consisting of cyclophosphamide, busulfan, fludarabine, thiotepa, and rabbit anti-thymocyte globulin. On January 8, 2026, he underwent transplantation from a 12/12 HLA-matched unrelated donor peripheral blood stem cell graft, with CD34+ cells at 9.50×10^6/kg, total nucleated cells at 7.97×10^8/kg, and CD3+ cells at 27.21×10^7/kg. GVHD prophylaxis included methotrexate, cyclosporine A, mycophenolate mofetil, and cyclophosphamide. Neutrophil and platelet engraftment both occurred on day +14. Donor chimerism on day +21 was 99.97%. The patient was discharged on day +25 (**Tables 2**, **3**).

### Patient 6

A 3-year and 4-month-old male patient was admitted on February 27, 2026. He developed jaundice shortly after birth, and repeated blood tests showed severe anemia with a nadir of 4.5 g/dL. Genetic testing confirmed congenital pyruvate kinase deficiency with compound heterozygous PKLR mutations (paternal c.730G>A p.G244R, maternal c.1054C>T p.P352S) (**Table 1**). He required regular red blood cell transfusions every 40–60 days. Pre-transplant serum ferritin was 1191.50ng/ml, and he received iron chelation therapy with deferasirox. Ultrasound and CT imaging revealed no splenomegaly. No other PKD-related complications, such as gallstones, were observed. He received a myeloablative conditioning regimen consisting of cyclophosphamide, busulfan, fludarabine, thiotepa, and rabbit anti-thymocyte globulin. On March 11, 2026, he underwent transplantation from a 9/10 HLA-matched unrelated donor peripheral blood stem cell graft, with CD34+ cells at 7.60×10^6/kg, total nucleated cells at 8.41×10^8/kg, and mononuclear cells at 6.81×10^7/kg. GVHD prophylaxis included methotrexate, cyclosporine A, and mycophenolate mofetil. At 3 months post-transplant, the patient remained clinically stable, had achieved complete transfusion independence, maintained full donor chimerism (**Tables 2**, **3**).

## Discussion

The prevalence of PKD is estimated at 3–8 per 1000000 (14, 15). Given its rarity, challenges in the diagnosis, and the wide-ranging spectrum of clinical symptoms, many cases may undiagnosed. Patients present variable clinical features due to chronic hemolysis and its complications, ranging from unrecognized mild compensated anemia to severe hyperbilirubinemia and anemia requiring exchange transfusions (11, 16), which makes early diagnosis of PKD difficult. Advances in gene sequencing have improved the sensitivity and accuracy of diagnosis (17).

In recent years, studies on PKD have made promising strides. More than 300 mutations in *PKLR* have been described, and most patients have at least one missense mutation. Splenectomy is considered effective for preventing active erythrocyte destruction, but the requirement for blood transfusions may not be completely eliminated as hemolysis persists after splenectomy (18, 19). Given the limitations of supportive treatments, increasing worldwide attention has arisen in genetic therapies (20, 21). Mitapivat, an oral allosteric activator of wild-type and mutant pyruvate kinase enzymes, has been approved by the FDA for hereditary PKD in adults (22). However, previous studies reported a lack of response to Mitapivat in several patients with certain genetic mutations (23). Patients with two non-missense mutations or who were homozygous for the R479H mutation showed a low likelihood of response. Further studies on pyruvate kinase activators are needed across broader genotypes. HSCT is a curative option for PKD eradication (24–26), but limited availability of histocompatible donors and serious complications associated with bone marrow transplantation in these patients (i.e., GVHD, hepatic veno-occlusive disease, hemorrhagic cystitis, poor graft function, opportunistic infections, engraftment syndrome, iron overload, etc.) limit its application. In a previous global cohort study (27), 16 PKD patients received transplants in Europe and Asia. The results showed Grade 3–4 graft-versus-host disease (GVHD) in a large group of patients (44%), with 5 of 16 patients (31%) dying from GVHD. The 2-year cumulative survival was 74%. High risk of morbidity and mortality presents an obstacle for HSCT, but with advances in clinical medicine and supportive care, the survival rate has significantly improved.

The present case series of six pediatric PKD patients undergoing HSCT demonstrates superior outcomes compared to historical data, with 100% survival and no severe GVHD observed, which contrasts with the 74% 2-year survival rate reported in previous studies (28). Notably, our cohort exhibited faster engraftment (median 12–16 days) and higher donor chimerism (>98% by day 21) than typical PKD transplant recipients. This may be attributable to our optimized conditioning regimen combining busulfan, cyclophosphamide, fludarabine, and thiotepa (29). Furthermore, the absence of severe GVHD reflects our modified prophylaxis protocol. Although CsA and MMF are recognized as standard GVHD prophylaxis globally (30), relying solely on this dual combination is often insufficient for patients receiving unrelated donor HSCT. A notable feature of this series is the consistent use of a thiotepa- and anti-thymocyte globulin (ATG)–based conditioning backbone across all patients. Thiotepa has been increasingly incorporated into conditioning strategies for non-malignant pediatric disorders due to its potent myeloablative and immunoablative properties, while ATG provides effective *in vivo* T-cell depletion that facilitates engraftment and reduces alloimmune activation (31–33). Previous studies in pediatric non-malignant diseases have suggested that thiotepa-containing regimens combined with busulfan, fludarabine, and ATG are associated with high engraftment rates and acceptable toxicity profiles, which is consistent with the outcomes observed in our cohort. Although direct comparisons are limited by differences in patient populations and study design, the uniformly rapid hematologic recovery and absence of primary graft failure in our series support the feasibility of this conditioning platform in PKD. Additionally, short-course MTX was routinely added to the CsA and MMF backbone to provide synergistic early immunosuppression. Notably, for older patients at higher risk of immune complications, post-transplant cyclophosphamide (PTCy) was integrated into the regimen. This tailored combination strategy effectively curtailed alloreactive T-cell responses, contributing to favorable GVHD-free outcomes.

Importantly, pre-transplant disease burden, including long-standing transfusion dependence, elevated ferritin levels indicative of iron overload, and splenomegaly in selected patients, did not appear to adversely affect engraftment kinetics in this cohort. All patients achieved timely neutrophil and platelet recovery regardless of baseline iron status or splenic size. This observation is in line with reports from other non- malignant hematologic disorders suggesting that ferritin level and splenomegaly are not absolute determinants of engraftment success when adequate myeloablative conditioning is applied (impact of iron overload on hematopoietic stem cell transplantation in pediatric patients with nonmalignant hematological disorders). It is plausible that the intensity of the conditioning regimen in this series overcame potential inhibitory effects of iron overload and altered marrow microenvironment, thereby enabling robust donor hematopoietic reconstitution.

The successful HSCT outcomes in our pediatric PKD cohort underscore several critical points regarding transplant timing and genotype-phenotype correlations. The progressive transfusion dependence observed in all patients, rising from intervals of every 1.5–3 months to monthly prior to HSCT, highlights the importance of early intervention before irreversible iron overload or alloimmunization develops. This is supported by recent studies in other transfusion-dependent disorders showing that transplantation performed before 10 years of age is associated with superior outcomes (34). The technical success of our protocol, achieving 100% donor chimerism without GVHD, validates recent advancements in conditioning regimens and stem cell processing. In particular, the use of thiotepa enhances marrow ablation and peripheral blood stem cells facilitate rapid engraftment. These results align with emerging data suggesting that genotype-stratified approaches can optimize transplant outcomes, where severe genotypes may benefit from more intensive conditioning, while compound heterozygotes require balanced regimens. Importantly, our experience supports the development of clinical decision trees incorporating both genetic and clinical parameters, as the integration of molecular diagnostics with transplant timing has been shown to significantly improve long-term survival in other rare hematologic disorders (35).

When comparing the pre-transplant clinical status of our current cohort to historically published cohorts, a distinct shift in the clinical paradigm is evident. In a previous global cohort study encompassing 16 PKD patients (27), HSCT was predominantly reserved as a “last resort” for older patients who had already suffered from massive iron overload, alloimmunization, and irreversible end-organ damage, with the majority having previously undergone splenectomy. Consequently, that historical cohort experienced high rates of severe Grade 3–4 GVHD and a 2-year cumulative survival of only 74%. In contrast, our cohort predominantly consists of young pediatric patients who underwent transplantation early in their disease course, with preserved organ function, intact spleens, and insignificant extramedullary hematopoiesis. The excellent overall survival (100%), high engraftment rates, and absence of severe GVHD observed in our study underscore the significant advantages of this proactive approach. Therefore, our findings support consideration of early HSCT for severe PKD before irreversible complications arise. Based on our clinical experience and the outcomes of this study, we propose the following indications for early HSCT in PKD: (1) Transfusion dependence: Frequent requirement of red blood cell transfusions that impairs the child’s normal growth and quality of life; (2) Progressive iron overload: Inadequate response to or intolerance of iron chelation therapy, leading to rising ferritin levels or early signs of organ deposition; (3) Avoidance of splenectomy: HSCT should ideally be performed prior to the clinical necessity of splenectomy to mitigate lifelong risks of severe infections and thrombotic events; (4) Availability of a suitable donor: Utilizing matched sibling or well-matched unrelated donors early in life, before significant alloimmunization occurs.

However, these promising results must be interpreted cautiously given the small sample size and relatively short follow-up period. Additionally, while our findings suggest genotype-specific responses, the limited number of cases may preclude definitive conclusions about optimal transplantation strategies for different mutation types. These limitations underscore the need for larger multicenter studies with extended follow-up to validate our observations and better characterize the risks and benefits of HSCT in pediatric PKD. Limited by the follow-up duration, the current study mainly focused on the short-term prognosis after HSCT and more studies are needed to analyze the long-term outcomes after HSCT, such as chronic GVHD, endocrine dysfunction, fertility outcomes, growth and developmental effects, secondary malignancies. Future research should also explore reduced-intensity conditioning regimens to minimize long-term toxicity while maintaining efficacy. Despite these constraints, our experience provides valuable real-world evidence supporting HSCT as a potentially curative option for carefully selected pediatric PKD patients, particularly those with severe phenotypes or genotypes less likely to respond to emerging pharmacotherapies. Furthermore, our findings suggest that HSCT represents an effective therapeutic approach not only for PKD but also for other severe congenital hemolytic disorders with unfavorable genotypes poorly responsive to pharmacotherapy, such as β-thalassemia and sickle cell disease. Therefore, the development of standardized transplantation protocols and risk stratification tools will be essential to guide clinical decision-making for these rare inherited diseases.

## Conclusions

In this study, all 6 patients achieved successful engraftment, high donor chimerism, and survived without severe complications. Optimized myeloablative HSCT provides a safe, effective, and curative treatment for pediatric patients with transfusion-dependent PKD. Our study suggests that HSCT represents an effective therapeutic approach for PKD and other hemolytic disorders with unfavorable genotypes poorly responsive to pharmacotherapy, such as β-thalassemia and sickle cell disease. Further studies are needed to improve the therapeutic efficacy and long-term prognosis of HSCT, as well as to reduce the incidence of complications.

## Data Availability

The original contributions presented in the study are included in the article/supplementary material, further inquiries can be directed to the corresponding author/s.
